# Plasma treatment of ZnO tetrapod–BiOBr heterojunction supported on PET waste for photocatalytic degradation of oil in water

**DOI:** 10.1038/s41598-025-32882-2

**Published:** 2025-12-27

**Authors:** Fahimeh Nourabi, Somaiyeh Allahyari, Nader Rahemi, Yogendra Kumar Mishra

**Affiliations:** 1 Chemical Engineering Faculty, Sahand University of Technology, P.O.Box 51335- 1996, Sahand New Town, Tabriz, Iran; 2 Fuel and Energy Research Laboratory, Sahand University of Technology, P.O.Box 51335-1996, Sahand New Town, Tabriz, Iran; 3 Smart Materials, NanoSYD, Mads Clausen Institute, University of Southern Denmark, Alsion 2, 6400 Sønderborg, Denmark

**Keywords:** ZnO tetrapod, Photocatalyst, Plasma treatment, Oily wastewater, Recycled polyethylene terephthalate, Chemistry, Environmental sciences, Materials science, Nanoscience and technology

## Abstract

In this study, a ZnO tetrapod–BiOBr heterojunction was ultrasonically immobilized onto floating recycled polyethylene terephthalate (PET) at different weight ratios (5, 10, and 15%) and evaluated for the photocatalytic degradation of hexane as a refractory oily pollutant under simulated solar light. The ZnOT(5)-B/P composite exhibited outstanding performance, achieving 96.6% degradation within 40 min due to its high surface area, strong ZnO–BiOBr interfacial contact, and efficient visible-light utilization. Comprehensive characterization (XRD, UV–Vis DRS, FESEM, BET/BJH, PL, AFM, CV, FTIR, Mott Schottky, ICP, and WCA) confirmed the formation of highly crystalline ZnO tetrapods and BiOBr microspheres with robust adhesion to the PET substrate and favorable bandgap reduction (3.1 → 1.8 eV), promoting enhanced charge separation. In the subsequent phase, the ZnOT(15)-B/P sample, which initially exhibited the lowest activity, was subjected to air-plasma surface engineering to address its weaker interfacial and textural features. Post-treatment structural and surface analyses (FESEM, FTIR, CV, and WCA) revealed that plasma exposure generated nanoscale surface etching, increased roughness and interparticle connectivity, and improved hydrophobicity, thereby facilitating stronger hexane adsorption and accelerated interfacial charge transfer. Furthermore, CV and XPS results indicated enhanced redox kinetics and increased density of surface oxygen vacancies, while AFM showed a ≈ 2.3-fold increase in surface roughness. As a result, the photocatalytic performance of ZnOT(15)-B/P improved substantially, rising from 67.5% to 84.8%, demonstrating the effectiveness of plasma post-activation in restoring and boosting the activity of an initially underperforming photocatalyst.

**Supplementary Information:**

The online version contains supplementary material available at 10.1038/s41598-025-32882-2.

## Introduction

Oily wastewater is a significant byproduct produced by a range of sources including refineries, oil wells, petrochemical plants, gas stations, transportation vehicles, and leaking pipelines^1,2^. Various treatment methods for oily wastewater have been developed, such as evaporation, gravity separation, flotation, flocculation, and filtration^3,4^. However, these methods tend to have low efficiency when used in isolation, making it more effective to combine them with additional techniques for improved performance^5^.

Heterogeneous photocatalysis, a type of advanced oxidation process (AOP), has been extensively researched and shown to be effective in completely degrading persistent and non-biodegradable pollutants in oily wastewater^6^. This process harnesses light energy to initiate redox reactions, generating highly reactive radicals such as hydroxyl and superoxide, which subsequently break down oily compounds into carbon dioxide and water^7–9^. Among the various photocatalysts available, zinc oxide (ZnO) has attracted significant attention. ZnO is a n-type semiconductor characterized by high photonic stability, environmental friendliness, and low cost. ZnO can exhibit a variety of morphologies, including flower-like nanostructures^10–14^, nanoplates^15–17^, and spherical nanoparticles^18^. Recently, ZnO tetrapods have been investigated due to their promising properties, such as a macroscopic three-dimensional flexible network, high porosity, substantial mechanical strength, enhanced surface area, and chemical durability^19^. However, its wide bandgap limits its efficacy under visible light. To address this limitation, several approaches have been explored, one of which involves the formation of heterojunctions between ZnO and other semiconductors to lower the bandgap^20–25^.

BiOBr acts as a p-type semiconductor with a distinctive layered hierarchical microstructure, efficient electron transport in composites^26^, and an indirect band gap^27^. When coupled with ZnO, it forms an internal electric field that promotes the separation of photogenerated charge carriers^28–30^.

In our previous work^30^, we employed a rapid microwave-assisted heating method to synthesize multipod ZnO, which was then combined with BiOBr to form a heterojunction for the photocatalytic degradation of oily wastewater. In the present study, however, we focus solely on ZnO “tetrapods” synthesized using the flame transport synthesis (FTS) method. Although this technique is energy-intensive and relatively slow, it yields highly pure ZnO tetrapods. Then ZnO tetrapod-BiOBr heterojunction with different percentages of ZnO tetrapod were embedded on recycled polyethylene terephthalate (r-PET) from drinking bottles as a floating substrate. Recently, floating photocatalysts have gained significant attention for their effectiveness in removing oily contaminants from water. Their capacity to efficiently absorb light, oxygen, and oil, along with the ease of subsequent collection, reduces catalyst loss and enhances their applicability in real-world scenarios. Common substrates used to create floating photocatalysts include perlite^31^, cork^32^, expanded graphite^33^, and lightweight polymers such as polypropylene^34^, polyethylene^35^, polystyrene^36^. These floating substrates typically exhibit several key characteristics: (1) durability under various environmental conditions, (2) hydrophobicity that attracts oil compounds to their surfaces, (3) cost-effectiveness and widespread availability, (4) low density, and (5) the ability to be molded into diverse shapes to optimize photocatalyst incorporation^37–39^.

The selection of PET in this study was motivated by both environmental and functional considerations. As one of the most widely used plastics, utilizing recycled PET contributes to plastic waste valorization. Moreover, PET is lightweight, chemically stable, and hydrophobic, enabling it to float effectively on the water surface and assist in the adsorption of oil. Characterizations such as X-ray diffraction (XRD), Field Emission Scanning Electron Microscopy (FESEM), N_2_ adsorption analysis (BET/BJH), Fourier-Transform Infrared Spectroscopy (FTIR), Water Contact Angle (WCA), UV–Vis Diffuse Reflectance Spectroscopy (UV–Vis DRS), Cyclic Voltammetry (CV), Thermogravimetry–Differential Thermogravimetry (TG-DTG) and Photoluminescence Spectroscopy (PL) were applied to find the effect of ZnO tetrapod percentage on the structure and thus activity of the resultant ZnO tetrapod-BiOBr/PET photocatalysts in degradation of hexane in water.

Applying non-thermal plasma (NTP) for the post-treatment of catalysts involves surface modifications that enhance catalytic activity. Literature studies indicate that plasma post-treatment of porous catalysts can disrupt the porous structure, leading to alterations in pore openings^40^. Additionally, NTP stimulates active phases by reinforcing them on the surface, resulting in improved inter-dispersion of active sites in supported catalysts^41^. This process enhances the stability of the catalyst compared to conventionally synthesized and untreated samples^42^ which is critical in our work due to weak bonding between inorganic photocatalyst particles of ZnO and BiOBr with the polymer substrate of PET. Moreover, the application of NTP yields improved morphology, increased surface area, enhanced pore size distribution, and a greater number of functional groups available for reactions^43^. For photocatalysts, changes in surface roughness, light absorption, and charge carrier recombination have also been documented^44^. Numerous studies have explored the effects of plasma treatment on TiO_2_ as a photocatalyst^20–22^; however, research on its influence on organic–inorganic photocatalysts, such as the one presented in this study, remains limited. In this context, the least efficient photocatalyst in the study was subjected to NTP treatment. The plasma-treated photocatalyst was then analyzed for changes in topology, morphology, hydrophobicity, functional groups, and voltammetric behavior to evaluate the impact of plasma treatment on its physicochemical properties. This investigation aimed to address whether a poorly performing photocatalyst can enhance its functionality through post-synthesis plasma modification, or if its performance is predominantly determined during the initial synthesis process.

## Materials and methods

### Materials

Zinc powder (particle size ≈ 10 µm, 99.99%), potassium bromide (KBr, 99%), bismuth nitrate pentahydrate (Bi(NO_3_)_3_·5H_2_O, 99%), ethylene glycol (EG, (HOCH_2_)_2_), dimethyl sulfoxide (DMSO), and ethanol (C_2_H_5_OH, 99%) were purchased from Merck. Polyvinyl butyral (PVB) was provided by Sigma-Aldrich. Polyethylene terephthalate (PET) was obtained from used water bottles collected from recycling bins in the university cafeteria. The *n*-hexane (Merck) was employed as the oil phase, and Span 20 (Merck) was used as the emulsifier for the preparation of the oil-in-water emulsion.

### Preparation of ZnO tetrapod-BiOBr/r-PET photocatalyst

#### Preparation of ZnO tetrapod

ZnO tetrapod was synthesized using the FTS method^45–47^. In this process, a crucible is filled with a mixture of Zn particles and PVB powder in a 1:2 ratio and then heated in a furnace that had been preheated to 450 °C in ambient air. Then the mixture was maintained at 900 °C for 30 min. At this elevated temperature, the PVB polymer burns, and the resulting flames convert Zn to white fluffy ZnO tetrapod powders^46^. After 30 min, the heating is switched off and the furnace is cooled down naturally followed by the tetrapods powder collection.

#### Preparation of BiOBr

BiOBr was synthesized via a simple solvothermal method. First, 0.8 g of Bi(NO_3_)_3_·5H_2_O and 0.15 g of KBr were dissolved separately in 15 mL of ethylene glycol and sonicated for 15 min. The KBr solution was then slowly added dropwise to the Bi(NO_3_)_3_·5H_2_O solution under continuous stirring. After stirring the mixture for an additional 30 min using a magnetic stirrer, the resulting suspension was transferred into a 100 mL Teflon-lined stainless-steel autoclave and heated at 150 °C for 12 h. After natural cooling to room temperature, the obtained precipitate was collected by filtration, washed twice with distilled water and once with ethanol, and finally dried at 60 °C for 12 h.

#### Recycling PET

Recycled PET used in this study was reclaimed from polyethylene terephthalate plastic bottles through a synthetic method described in earlier report^30^. First, used drink bottles were collected and cut into small pieces. These pieces were then mixed with DMSO in a 1:4 weight ratio. The mixture was stirred on a magnetic stirrer for 15 min at 180 °C. After mixing, the clear solution was quickly cooled using distilled water. To remove any remaining DMSO, the solution was washed multiple times with warm deionized water.

#### Immobilization of ZnO tetrapod-BiOBr heterostructure on recycled PET

ZnO–BiOBr/PET composites with varying ZnO contents (5, 10, and 15 wt%) were prepared by adding the corresponding amounts to separate beakers containing 100 mL of distilled water and 80 wt% recycled PET and BiOBr. The total weight percentage of ZnO tetrapods and BiOBr was maintained at 20 wt% in each formulation. The mixtures were sonicated for 15 min to achieve homogeneity and then placed in an oven at 100 °C for 24 h. The samples were designated as ZnOT(x)-B/P, where *x* represents the weight percentage of ZnO tetrapods in the composite and takes values of 5, 10, or 15 wt%. ZnOT(20)/P denotes the film comprising 20 wt% ZnO tetrapods on a PET substrate in the absence of BiOBr, while B(20)/P corresponds to the sample containing 20 wt% BiOBr on PET without ZnO tetrapods.

### Characterization of ZnO tetrapod-BiOBr/PET samples

The structural, morphological, and physicochemical properties of the synthesized ZnO Tetrapod–BiOBr/PET photocatalysts were characterized using the following techniques: Crystallographic analysis was conducted via X-ray diffraction (XRD, D8 ADVANCE, Bruker) using Cu Kα radiation (λ = 1.5406 Å) at a scanning rate of 0.06 s^−1^ within a 2θ range of 10°–70°, to determine the phase composition and crystallinity of the samples. Surface morphology and elemental mapping were observed using field emission scanning electron microscopy (FESEM, MIRA3, TESCAN) coupled with energy-dispersive X-ray spectroscopy (EDX), providing detailed insight into the microstructure and elemental distribution. Textural properties including specific surface area were evaluated using nitrogen adsorption–desorption measurements (ChemBET 3000). The surface area was calculated via the Brunauer–Emmett–Teller (BET) method. Functional groups were identified through Fourier-transform infrared spectroscopy (FTIR, Perkin Elmer), recorded in the range of 4000–400 cm^−1^ with a resolution of 4 cm^−1^, to confirm chemical bonding features. Wettability was assessed by static water contact angle measurements at room temperature using a goniometer (Jikan CAG-20 PE), allowing determination of hydrophobicity/hydrophilicity of the surface. Optical absorption characteristics were analyzed using UV–Vis diffuse reflectance spectroscopy (DRS, SCINCO S-4100), and the optical band gaps were estimated using the Kubelka–Munk function. Photoluminescence (PL) spectra were recorded at an excitation wavelength of 325 nm with a Varian fluorescence spectrophotometer to evaluate the recombination behavior of photogenerated charge carriers. Electrochemical analysis was performed using a three-electrode cell connected to a SAMA500 electroanalyzer. The working electrode was prepared by coating ZnO Tetrapod–BiOBr onto nickel foam, while a platinum wire and Ag/AgCl electrode served as counter and reference electrodes, respectively. All measurements were conducted in 1 M KOH aqueous electrolyte under ambient conditions. Atomic Force Microscopy (AFM) was 137 utilized to investigate the surface topography of the samples with a Digital Instruments 138 Nanoscope TM 3D ADC5 Multimode (Veeco Instruments Inc.). Mott–Schottky measurements were performed using an Ivium Vertex electrochemical workstation in a conventional three-electrode configuration. A glassy carbon electrode was used as the working electrode, platinum wire served as the counter electrode, and a saturated Ag/AgCl electrode was employed as the reference. The electrolyte consisted of 0.1 M Na_2_SO₄ aqueous solution. The measurements were recorded under a constant applied current of 10 mA with an AC perturbation frequency of 2 kHz, over a potential range from + 1.0 to − 1.0 V (vs. Ag/AgCl).

### Photocatalytic degradation of oil in water emulsion

The photocatalytic degradation of *n*-hexane in an oil-in-water emulsion was systematically investigated under visible light irradiation. Prior to illumination, the photocatalyst was dispersed into the emulsion and magnetically stirred in the dark for 10 min to achieve adsorption–desorption equilibrium on the catalyst surface. The photoreactor was equipped with two 150 W tungsten halogen lamps (OSRAM, Poland), providing an irradiance of approximately 8000 lx at the sample interface. The initial hexane concentration was fixed at 1 vol%. Stable emulsions were prepared by homogenizing deionized water, *n*-hexane, and Span 20 surfactant, ensuring uniform dispersion of the oil phase. Photodegradation tests were carried out at a controlled temperature of 25 °C over a 40 min irradiation period. Upon completion, photocatalyst particles were separated via centrifugation at 10,000 rpm for 5 min. Hexane concentrations were periodically monitored by withdrawing 5 mL aliquots for UV–Visible spectrophotometric analysis. Catalyst recyclability was assessed by recovering the used photocatalyst through filtration, rinsing with distilled water, drying overnight at 100 °C, and reapplying it in subsequent cycles. The degradation (%) was quantified using the expression:

$${\mathrm{Degradation}}\left( \% \right)={{\mathrm{C}}_0} - {{\mathrm{C}}_{\mathrm{t}}}/{{\mathrm{C}}_0} \times {\mathrm{1}}00$$

where C_0_ indicates the initial concentration of *n*-hexane in the emulsion, and C_t_ represents the concentration of *n*-hexane at the times of irradiation.

To identify the dominant reactive species involved in the degradation mechanism, isopropanol (IPA) was employed as a scavenger for hydroxyl radicals (·OH), Acid Boric for superoxide radicals (·O_2_^−^), and ethylenediaminetetraacetic acid (EDTA) for photogenerated holes (h^+^).

### Plasma treatment of ZnOT(15)-B/P

To investigate the influence of non-thermal plasma treatment on the catalyst structure, the ZnOT(15)-B/P sample, identified as the least active photocatalyst, was exposed to dielectric barrier discharge (DBD) plasma at 80 kV for 5 min in ambient air. The treatment was conducted in a DBD plasma reactor equipped with stainless-steel electrodes spaced 5 mm apart.

## Results and discussions

### Characterization of ZnO tetrapod-BiOBr/PET photocatalysts

#### FESEM analysis

**Fig. 1:**
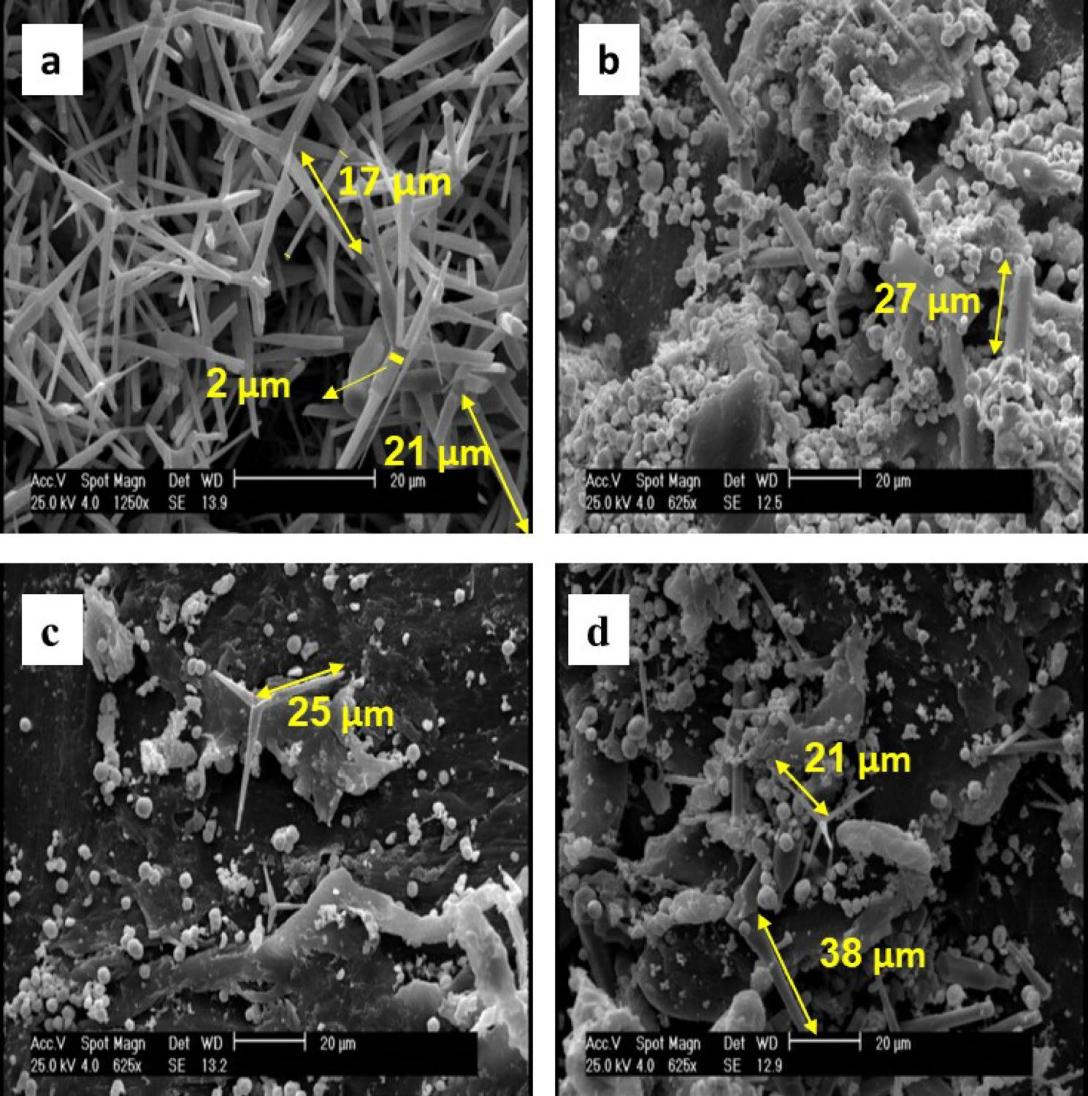
FESEM micrographs of (**a**) ZnOT, (**b**) ZnOT(5)-B/P, (**c**) ZnOT(10)-B/P, and (**d**) ZnOT(15)-B/P.

The morphology of synthesized ZnO tetrapods (ZnOT) and ZnOT(5)-B/P, ZnOT(10)-B/P, and ZnOT(15)-B/P was examined using FESEM, as shown in Fig. 1. The interconnected 3D ZnO tetrapods, characterized by long and sharp arms that contribute to a porous structure, were observed, consistent with findings reported in^46,48^. The synthesized ZnO tetrapods exhibited a base diameter of approximately 3–5 µm, tapering to a tip diameter of about 1 µm. The length of the tetrapod arms ranged from 15 to 20 µm. Each tetrapod arm was divided into three distinct regions: an initial section with a uniform diameter, a middle segment marked by an abrupt change in thickness, and a final tapered, needle-like end. As illustrated in Fig. 1S, the BiOBr microspheres (20 μm diameter) were composed of intertwined BiOBr plates with a thickness of around 20 nm similar to^44^. Fractured arms of ZnO tetrapods were observed, likely resulting from the ultrasonic waves applied during the immobilization of the ZnO Tetrapod–BiOBr composite into the PET substrate. This effect was particularly pronounced in the ZnOT(5)-B/P sample. Notably, in the ZnOT(5)-B/P sample, the ZnO tetrapods appeared to be embedded beneath the BiOBr microspheres, indicating a strong interaction between the components. However, the interactions between ZnO and BiOBr diminished in the ZnOT(10)-B/P and ZnOT(15)-B/P samples. Moreover, it is evident that BiOBr is present in an agglomerated form in ZnOT(5)-B/P, but as the amount of ZnO tetrapod increases (and the amount of BiOBr decreases, maintaining a total of 20% wt.), the BiOBr becomes more evenly dispersed.

#### XRD analysis

**Fig. 2:**
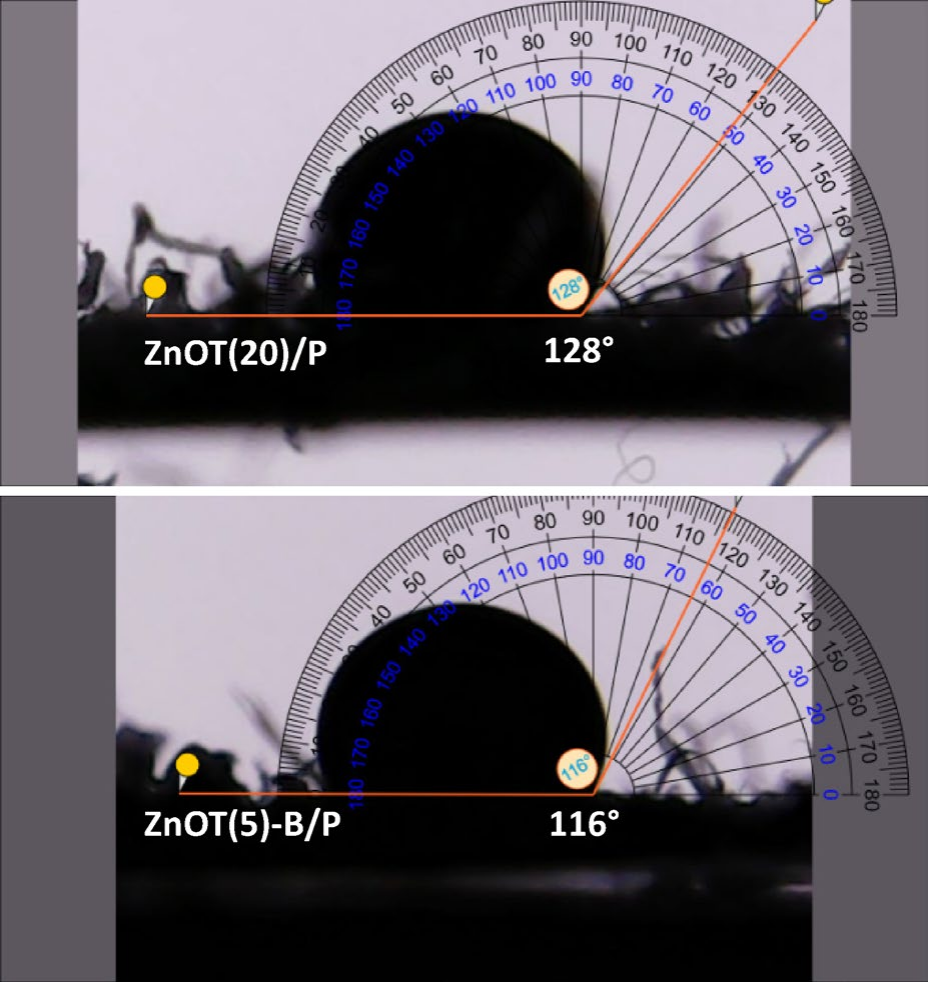
The water contact angle of ZnOT(20)/P and ZnOT(5)-B/P.

XRD was conducted to identify the crystalline phases present in the ZnOT(5)-B/P, ZnOT(10)-B/P, and ZnOT(15)-B/P samples, which were collected within the 2θ range of 10° to 70° and are presented in Fig. 2S. The XRD pattern of PET reveals a prominent peak at 2θ = 17°, 18°, 23.4°, and 25.8°^49,50^. Recycled PET used in our samples produced similar diffraction peaks, indicating that the crystalline structure of the PET was maintained after the recycling process. In all samples hexagonal wurtzite phase (JCPDS card No. 01-075-1526) was observed^51^. Additionally, presence of BiOBr is confirmed according to JCPDS No. of 00-009-0393^52^. In essence, as the amount of ZnO increases, the intensity of the ZnO peaks rises correspondingly, confirming a greater immobilization of ZnO in the ZnO–BiOBr-PET samples. The sharp and narrow diffraction peaks of ZnO indicate that the samples exhibit a high degree of crystallinity. The intensity of the BiOBr-related peak decreased from the ZnOT(5)-B/P to the ZnOT(15)-B/P samples, confirming a reduction in the BiOBr content, as suggested by the synthesis ratios.

#### BET/BJH analysis

**Fig. 3:**
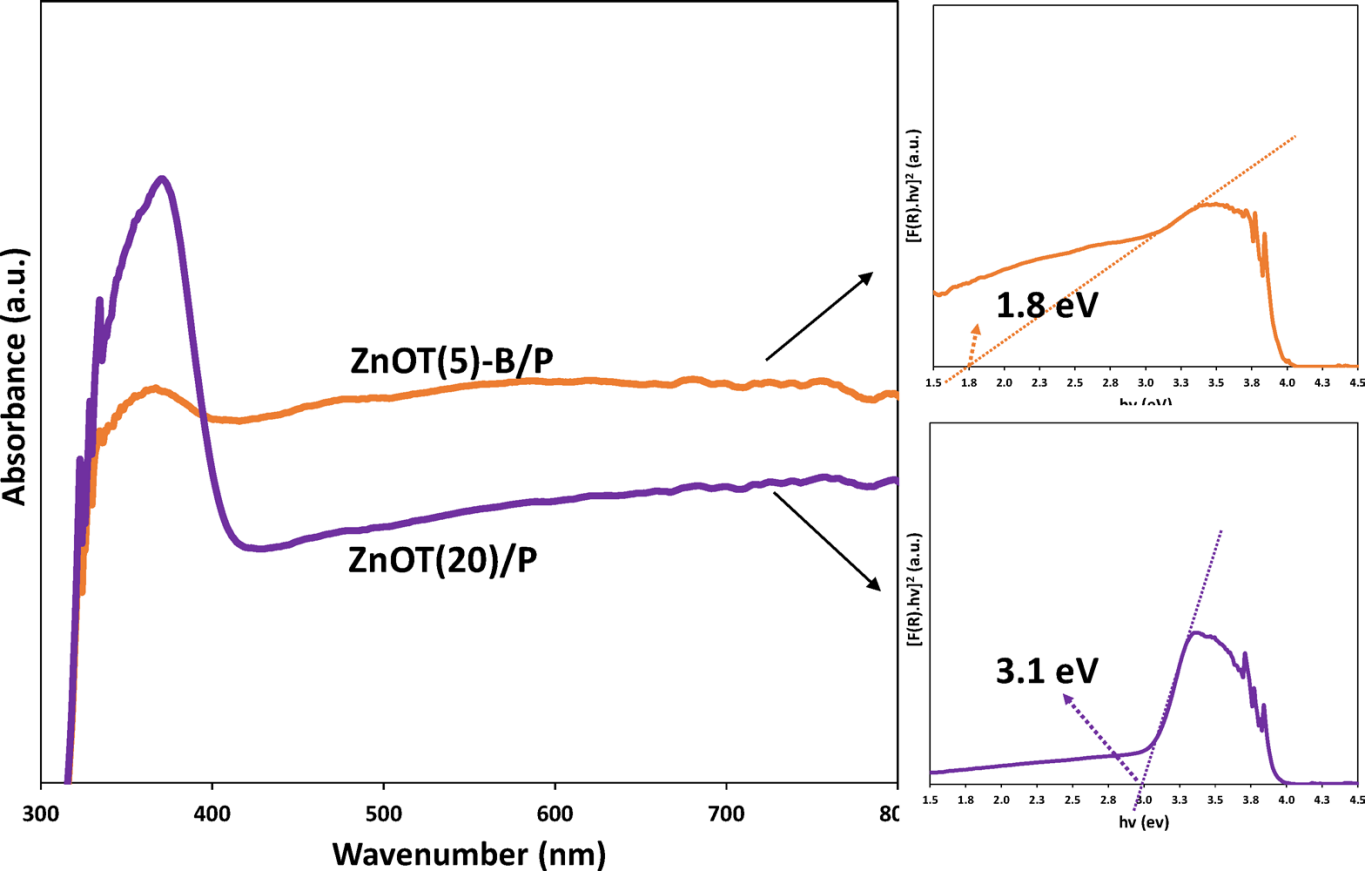
UV–Vis DRS of ZnOT(20)/P and ZnOT(5)-B/P and their Tauc plots.

The N_2_ adsorption/desorption isotherms for the ZnO Tetrapod-BiOBr/PET photocatalyst, differing in ZnO percentages, are illustrated in Fig. 3S and specific surface area and porous characteristics summarized in Table 1S. All samples exhibit type IV isotherm behavior, indicating a predominantly mesoporous structure^53,54^, according to International Union of Pure and Applied Chemistry (IUPAC) classifications. The observed H3 hysteresis type suggests the presence of wedge-shaped pores created by the loose stacking of 2D lamellar structures, commonly found in materials such as BiOBr^55^, PET^56^, or ZnO tetrapod^19^. The increasing content of ZnO tetrapods in the samples affected the adsorption isotherms, shifting nitrogen uptake to higher relative pressures, which indicates a decrease in overall porosity.

The inset of Fig. 3S presents the pore size distribution of the samples, demonstrating an increase in the population of micropores with the addition of ZnO tetrapods. Notably, the ZnOT(5)-B/P sample exhibited a considerable presence of macropores, exceeding 40 nm in size. As detailed in Table 1S, enhancement in ZnO tetrapods content decreased the porosity and surface area of the samples. Although the three-dimensional structure of ZnO tetrapods creates interstitial spaces among BiOBr aggregates and enhances their dispersion, as observed in FESEM images, the overall porosity decreases due to the predominance of the less porous ZnO tetrapods in the composite.

Among all the samples, ZnOT(5)-B/P exhibited the highest specific surface area (28.5 m2/g) and pore volume (0.095 cm^3^/g), attributed to its unique combination of micro- and macropores, despite the presence of agglomerated BiOBr. However, increasing the ZnO content to 15 wt% led to a reduction in both surface area and pore volume, with values decreasing to 19.5 m^2^/g and 0.065 cm^3^/g, respectively.

To specifically investigate the role of BiOBr in conjunction with ZnO tetrapods, the ZnOT(5)-B/P sample—exhibiting the highest surface area, porosity, and component interconnectivity—was compared to the ZnOT(20)/P sample, which contains no BiOBr in terms of WCA, DRS and PL analyses.

#### WCA analysis

Figure 2 shows that the water contact angle of the ZnOT(20)/P sample is 128°, indicating that this photocatalyst is hydrophobic due to the presence of PET, a hydrophobic material^57^. Polymeric materials and their components exhibit excellent water repellency and oil adsorption capacity, allowing for easy retrieval from the water surface^58^. This characteristic renders these photocatalysts especially suitable for enhancing adsorption and exhibiting a strong affinity for oily liquids^59^. The higher hydrophobicity of the ZnOT(20)/P sample can be attributed to the surface microstructures of ZnO tetrapods, which facilitate the repelling of water droplets. In this context, some studies appropriately utilize the Cassie-Baxter model to explain the behavior of this solid-air composite surface, as the microstructural design of the ZnO tetrapods traps air within their micro-sized arms^60,61^. This change is attributed to the replacement of ZnO tetrapods—characterized by their high surface roughness (as evidenced in FESEM images) and inherent hydrophobicity—with BiOBr, which exhibits a layered structure and relatively higher polarity. The presence of polar bromide ions and smoother morphology in BiOBr enhances the material’s interaction with water molecules, thereby lowering the contact angle. Nonetheless, despite the decrease to a contact angle of 116°, the composite remains hydrophobic, which is essential for its role as a floating photocatalyst with an affinity for oil adsorption.

#### UV–Vis DRS analysis

As illustrated in Fig. 3a, the binary photocatalyst ZnOT(20)/P demonstrated substantial light absorption, particularly in the UV region (below 400 nm), aligning with existing literature^62–64^. This corresponds to a bandgap of 3.1 eV, as indicated by the Kubelka–Munk plot. In contrast, the absorption edge of the ZnOT(5)-B/P shifted significantly toward longer wavelengths, reflecting a bandgap of 1.8 eV. This shift indicates enhanced visible light harvesting, which was a primary objective of this study. While the absorption in the UV region markedly decreased due to the lower proportion of ZnO in the ZnOT(5)-B/P , the capacity for visible light harvesting increased substantially. The noticeable variation in the band gaps between ZnOT(5)-B/P and ZnOT(20)/P originates from the formation of the ZnO/BiOBr heterojunction and the composition-dependent optical response of the samples. In ZnOT(5)-B/P, the incorporation of visible-light–responsive BiOBr and the interfacial band bending at the ZnO/BiOBr junction promote charge transfer and partial overlap of the electronic states, leading to a red-shift of the absorption edge and a narrower apparent band gap. However, when the ZnO content increases and BiOBr is absent (ZnOT(20)/P), the optical behavior is dominated by the intrinsic wide band gap of ZnO, which mainly absorbs in the UV region, resulting in a blue-shift and larger apparent band gap.

#### PL analysis

**Fig. 4:**
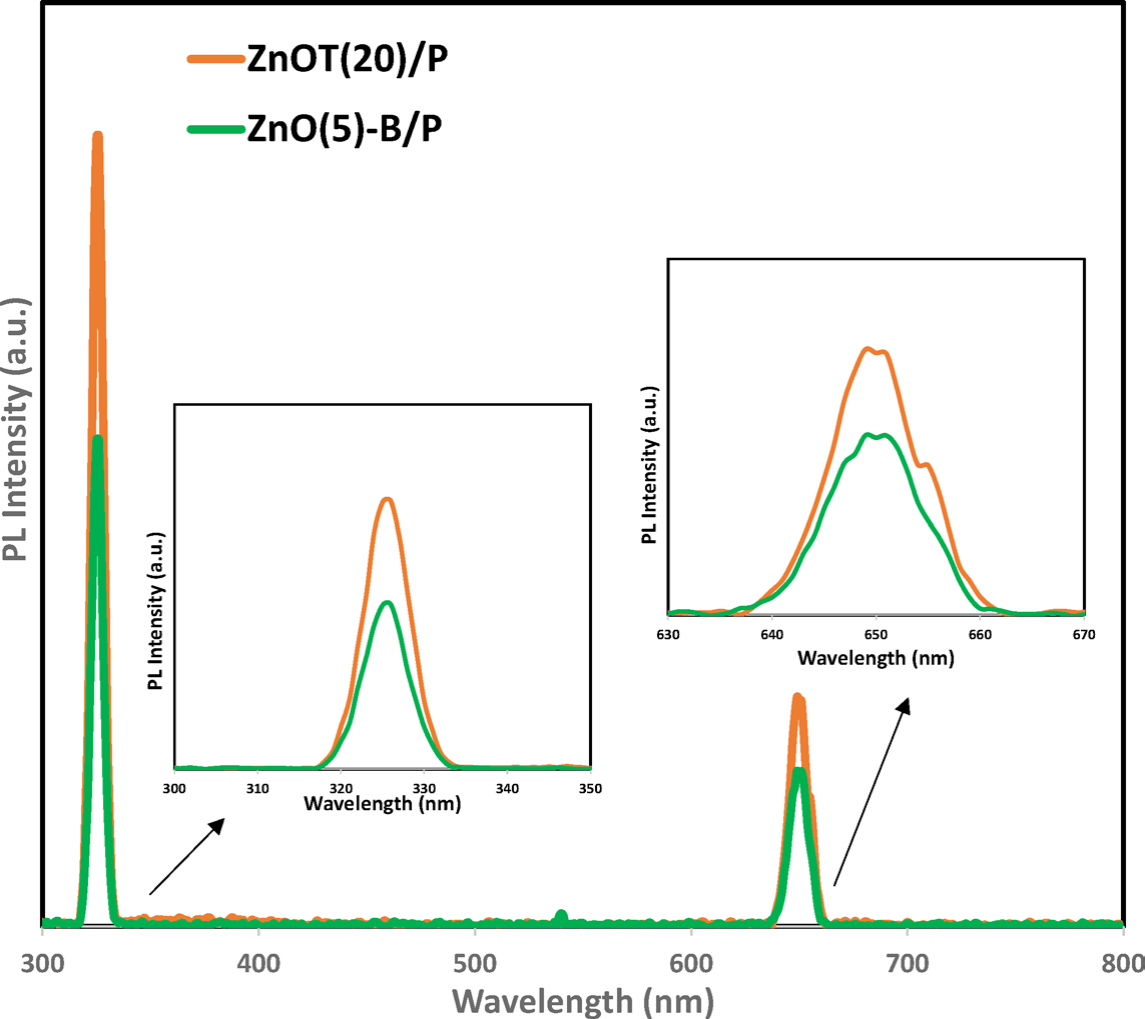
PL analysis of ZnOT(20)/P and ZnOT(5)-B/P.

Photoluminescence (PL) spectra for two samples—ZnOT(20)/P and 5ZnOT(5)-B/P—are presented in Fig. 4. Two types of PL peaks- near-band edge emission and deep-level emission were observed for both samples. The UV emission band (approximately 325 nm) is attributed to near band-edge transitions, specifically the free exciton recombination through an exciton-exciton collision process^65^. The reduced intensity of the UV emission in the ZnOT(5)-B/P sample indicates a lower rate of recombination between charge carriers. The intensity of the deep-level emission (600–700 nm) also decreases with the introduction of BiOBr, signifying a reduction in defects^66^. Reducing structural defects in photocatalysts generally improves performance by decreasing charge carrier recombination. However, some controlled defects can enhance activity by providing active sites, so an optimal balance is essential.

### Photocatalytic performance of ZnO Tetrapod-BiOBr-PET photocatalysts

The photocatalytic performance of the ZnOT(5)-B/P, ZnOT(10)-B/P, and ZnOT(15)-B/P photocatalysts was evaluated by assessing the degradation of a hexane-in-water emulsion under simulated sunlight irradiation (Fig. 6a). The experiments were conducted under specific conditions: 1% volume of hexane, pH 4.5, and 0.05 g of catalyst in 100 mL of solution. The selected operating parameters, including the hexane concentration (1% v/v), solution pH (4.5), and catalyst dosage (0.05 g per 100 mL), were determined based on systematic optimization experiments. The results of these optimization studies, showing the effects of hexane content, pH, and catalyst loading on the hexane degradation efficiency and stability, are provided in the Supplementary files (Figs. 6S–9S). The results indicated that the ZnOT(5)-B/P photocatalyst exhibited the highest activity, with a hexane degradation rate of 96.6%. FESEM analysis revealed a strong interaction between the ZnO tetrapods and BiOBr microspheres in this sample, enhancing the effectiveness of heterostructure formation. BET/BJH analysis demonstrated that the 5ZnOT(5)-B/P photocatalyst possesses an advantageous porous structure with a higher specific surface area, providing increased active sites for hexane degradation.

Moreover, the WCA measurements indicated the sample’s hydrophobic characteristics, suggesting it can easily adsorb oily compounds like hexane while repelling water. The ZnO tetrapods also form a 3D network with desirable porosity and mechanical strength by interconnecting their arms. This network effectively supports BiOBr, improving its dispersion on the PET substrate and enhancing visible light absorption^67,68^, as confirmed by FESEM and UV–Vis DRS analyses. Moreover, the presence of macropores in ZnOT(5)-B/P, as confirmed by BJH analysis, is significant, as it facilitates the diffusion of larger oil molecules and should not be overlooked.

**Fig. 5:**
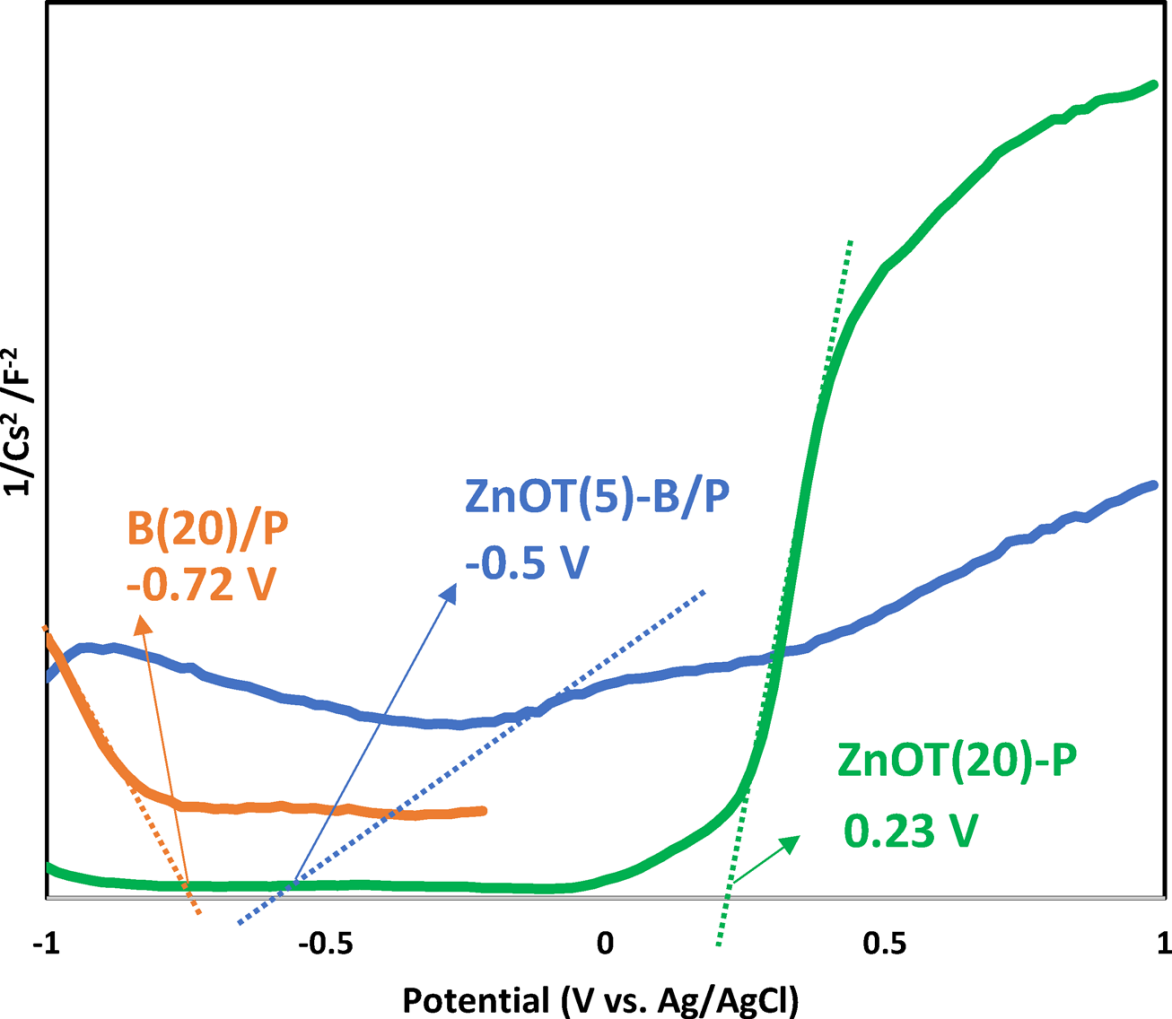
Mott-Schottky curves of ZnOT(20)/P, B(20)/P and heterojunction of ZnOT(5)-B/P.

When BiOBr and ZnO tetrapods form a composite, their Fermi levels align, resulting in a shift that creates an electric field at the interface between phases. To further verify the formation of an interfacial electric field between ZnO tetrapods and BiOBr, Mott–Schottky measurements were conducted for ZnO (ZnOT(20)/P), BiOBr (B(20)/P), and the ZnOT(5)-B/P heterojunction (Fig. 5). ZnO and the ZnO–BiOBr composite exhibit positive slopes in the linear region, indicating predominant n-type behavior arising from donor-type defects. In contrast, BiOBr presents a negative slope, confirming its p-type nature. The coexistence of n-type ZnO and p-type BiOBr therefore provides a rational basis for internal electric field formation in the heterojunction.

The flat-band potentials extracted from the linear regions were approximately − 0.50 V for ZnO, − 0.72 V for BiOBr, and + 0.23 V (vs Ag/AgCl) for ZnO–BiOBr. After conversion to the NHE reference, these correspond to conduction band positions of − 0.30 V and + 0.43 V for ZnO and ZnO–BiOBr, respectively. For p-type BiOBr, the flat-band potential reflects the valence band edge, yielding an estimated VB position (vs NHE) of + 0.48 V. Combining these potentials with the optical band gaps (3.1 eV for ZnO and 1.8 eV for ZnO–BiOBr), the valence band energies were calculated to be + 2.80 V and + 2.23 V vs NHE, respectively. Meanwhile, the conduction band of BiOBr was located at − 2.14 V vs NHE, obtained from VB–Eg.

This band configuration confirms the formation of a p–n heterojunction between ZnO and BiOBr, accompanied by significant band bending and charge redistribution at equilibrium. The internal electric field that develops at the interface facilitates migration of photogenerated electrons from the CB of ZnO toward BiOBr and drives hole accumulation within the BiOBr valence band. As a consequence, the heterostructure exhibits enhanced charge separation efficiency relative to the pristine materials, accounting for its improved photocatalytic performance.

The improved photocatalytic activity of ZnOT(5)-B/P under visible light can be attributed to its optical properties. Bandgap calculations revealed a decrease to 1.8 eV, attributed to the formation of a heterostructure between the ZnO tetrapod and BiOBr. This reduction suggests a significant increase in visible light response, leading to enhanced photocatalytic activity in this light spectrum.

In contrast, the photocatalytic activity of the other synthesized samples was lower, with degradation rates of 81.2%, and 67.5% for ZnOT(10)-B/P, and ZnOT(15)-B/P, respectively. The reduction in photocatalytic performance of the ZnOT(10)-B/P and ZnOT(15)-B/P samples is closely related to the weaker interfacial interactions between ZnO tetrapods and BiOBr microspheres at higher ZnO concentrations. FESEM images revealed that the dense packing of excess ZnO tetrapods reduces the exposure of BiOBr particles, while BET/BJH results (Table 1S) confirmed a decrease in surface area and pore volume with increasing ZnO content. This morphological change limits the effective heterojunction interface where charge separation occurs. Consequently, fewer active junctions are available for photoinduced electron–hole transfer. Therefore, at elevated ZnO loadings, agglomeration of tetrapods, partial coverage of BiOBr surfaces, and restricted charge transport collectively lead to the observed decline in interfacial coupling and photocatalytic activity.

**Fig. 6:**
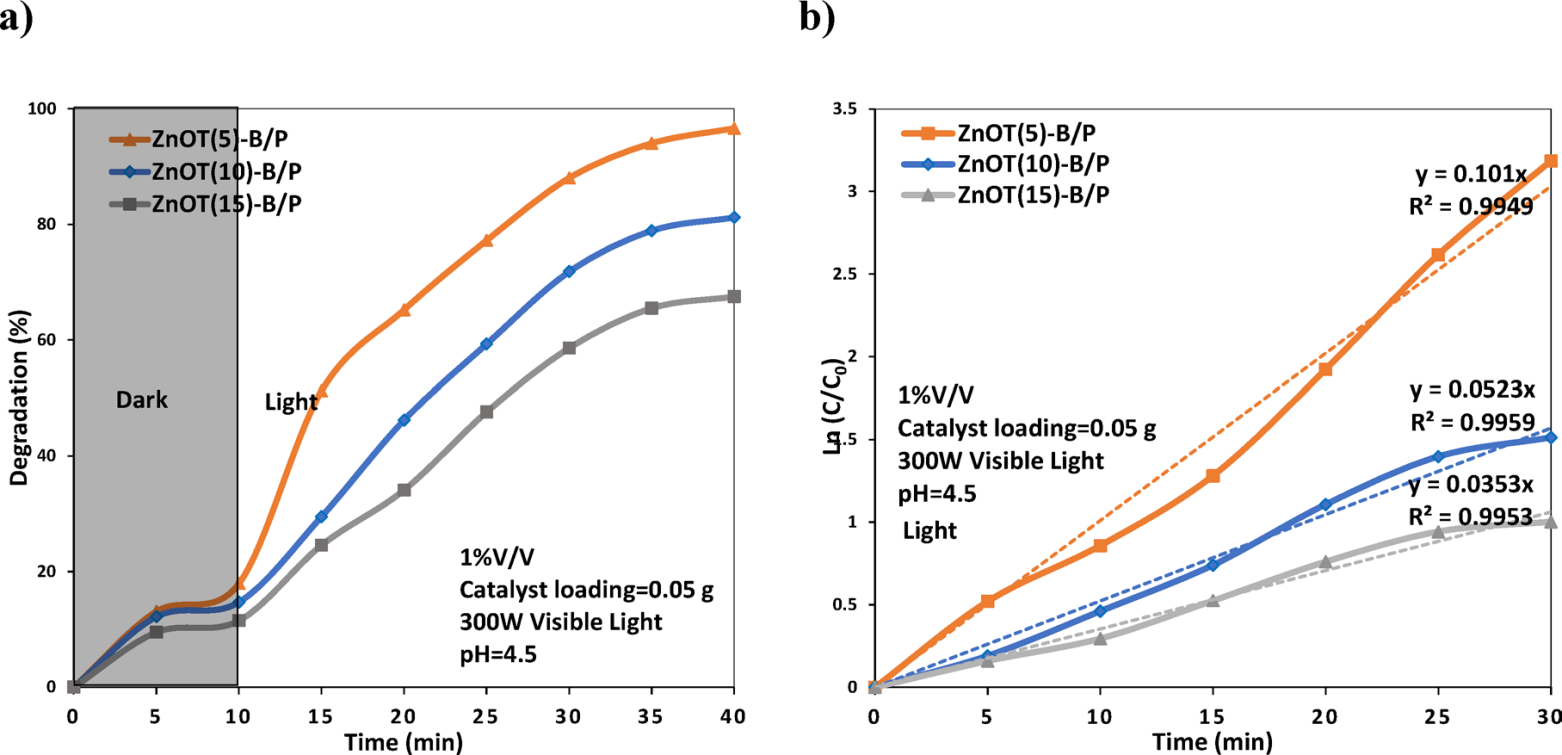
(**a**) Photocatalytic activity and (**b**) Kinetic study of ZnOT(x)-B/P samples in degradation of hexane in water.

Figure 6b, quantitatively investigated the kinetics of the reaction using a pseudo-first-order model. The rate constant for the ZnOT(5)-B/P sample was determined to be 0.101 (1/min), significantly higher than that of the other prepared photocatalysts. Specifically, the rate constants for ZnOT(10)-B/P, and ZnOT(15a)-B/P, were measured at 0.0523, 0.0353 (1/min), respectively.

**Fig. 7:**
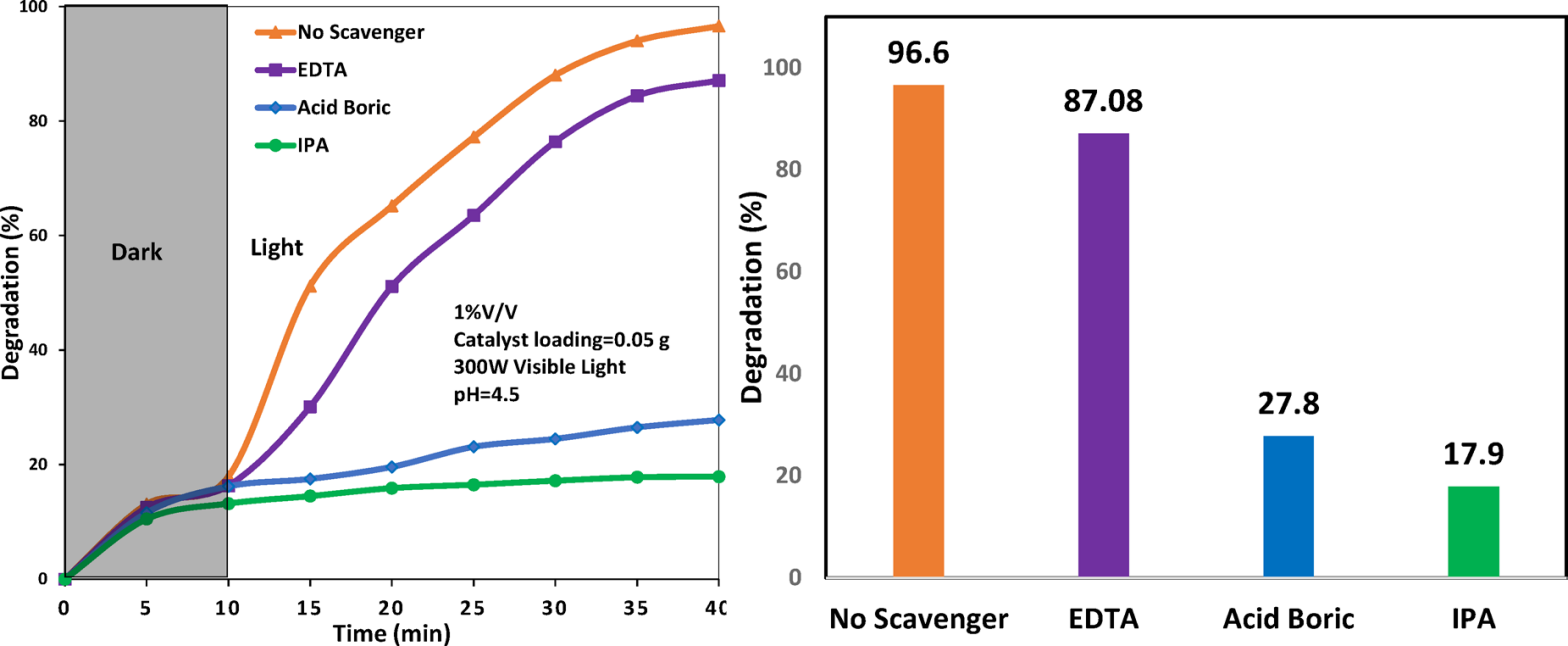
Scavenger test on ZnOT(15)-B/P photocatalyst in degradation of hexane in water.

Radical scavenging experiments (Fig. 7) according to^69^ revealed that the photocatalytic degradation efficiency decreased slightly in the presence of EDTA, indicating a minor contribution of photogenerated holes (h^+^). However, a dramatic decline was observed with boric acid (superoxide scavenger) and isopropanol (hydroxyl scavenger), suggesting that hydroxyl and superoxide radicals radicals play the significant role in the degradation process.

Figure 4S illustrates that pure PET experiences complete weight loss at relatively low temperatures during TGA analysis, whereas the incorporation of ZnOT(5)-B shifts the thermal decomposition to higher temperatures. Additionally, the non-zero residual weight confirms the successful embedding of ZnO tetrapod-BiOBr within the PET matrix—a key factor for ensuring the long-term stability of the composite, given the critical importance of organic–inorganic integration in this work.

### Plasma surface treatment of ZnOT(15)-B/P

**Fig. 8:**
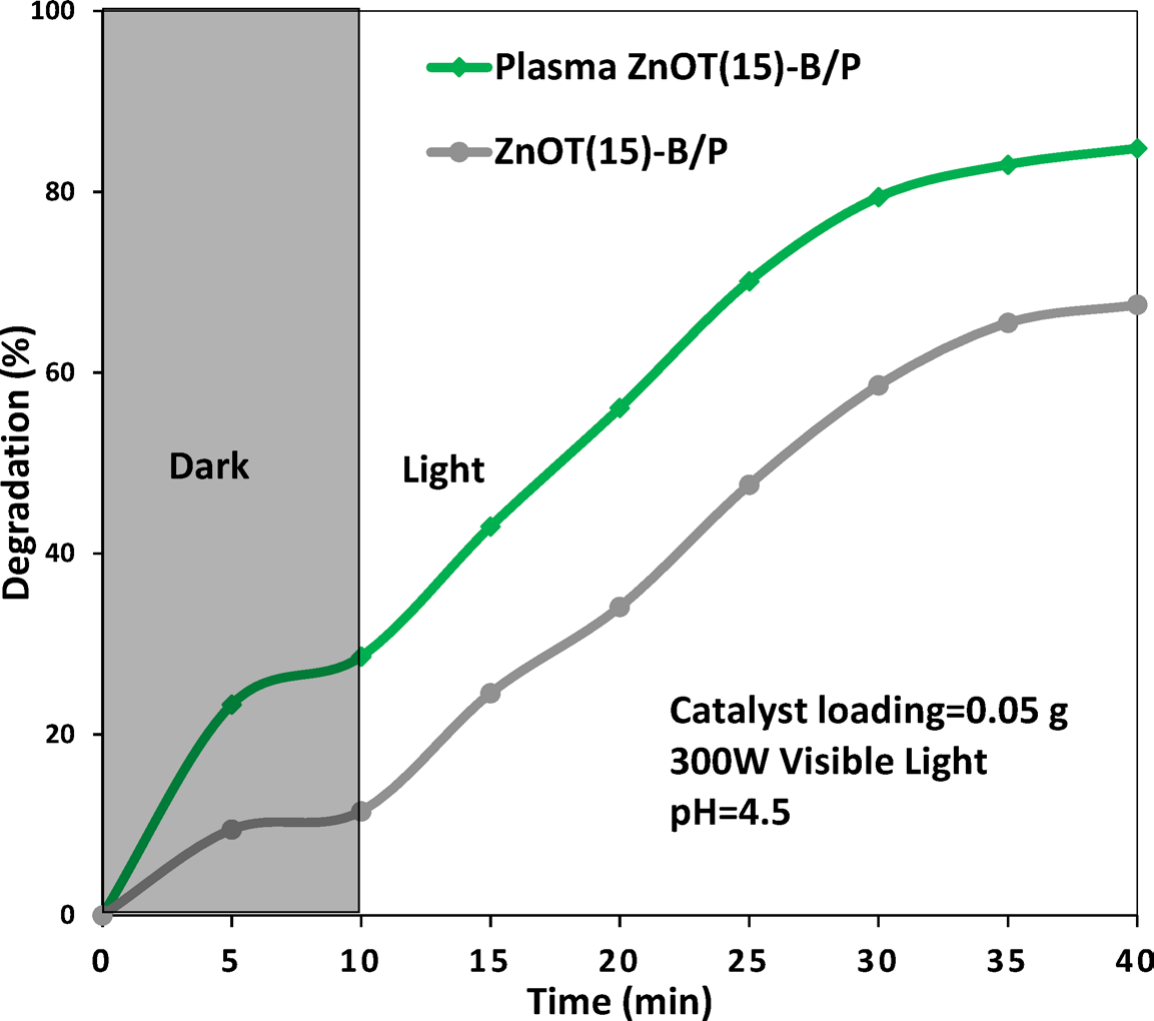
Activity of ZnOT(15)-B/P in degradation of hexane in water before and after plasma treatment.

**Fig. 9:**
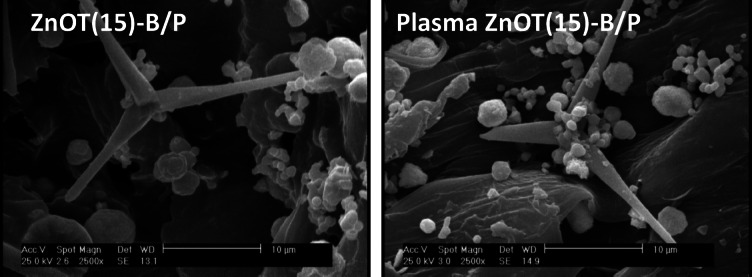
FESEM pictures of ZnOT(15)-B/P before and after plasma treatment.

In the second phase, we investigated the impact of air plasma treatment on ZnOT(15)-B/P photocatalyst which exhibited the lowest photocatalytic performance. Figure 8 illustrates that plasma treatment improved the performance of the treated catalyst in hexane degradation from a low amount of 67.5 to 84.8%. To find the reason, morphology of the sample before and after plasma treatment was investigated. Figure 9 presents a comparison of FESEM images for the ZnOT(5)-B/P before and after plasma treatment. As observed, the morphology of the ZnO tetrapods and BiOBr microspheres remained unchanged; however, the interactions between these two compounds and with PET were enhanced. This strengthened interaction is essential for photocatalytic reactions, as it facilitates the movement of electrons and holes toward other components, ensuring effective charge carrier separation—a critical characteristic of photocatalysts. Furthermore, the attachment to PET allows for the availability of hexane and oxygen, as PET serves as a hydrophobic component that adsorbs hexane while keeping the photocatalyst accessible to air.

**Fig. 10:**
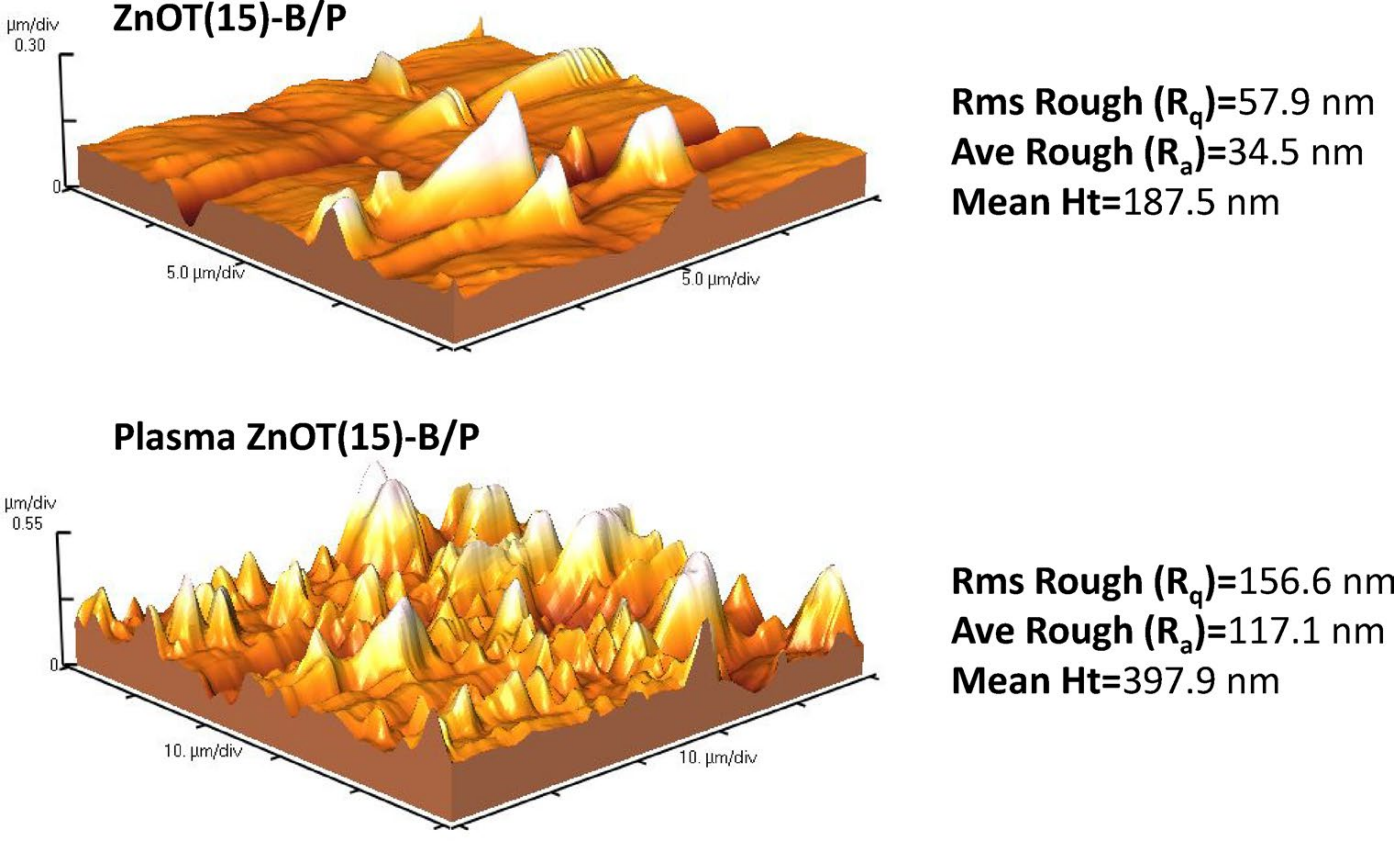
Surface topography of ZnOT(15)-B/P before and after plasma treatment.

Figure 10 displays the surface topography of the ZnOT(15)-B/P before and after plasma treatment as the roughness and homogeneity of the surface have been increased. The Root Mean Square (RMS) of the surfaces were calculated as 57.9 and 156.6 nm before and after plasma treatment, respectively. The increase in roughness indicates that plasma has detached particles from the surface and settled elsewhere^70^. Surface roughness increases the available surface area, resulting in a significant improvement in photocatalytic activity. Moreover, it provides more sites for the adsorption of hexane^71^.

**Fig. 11:**
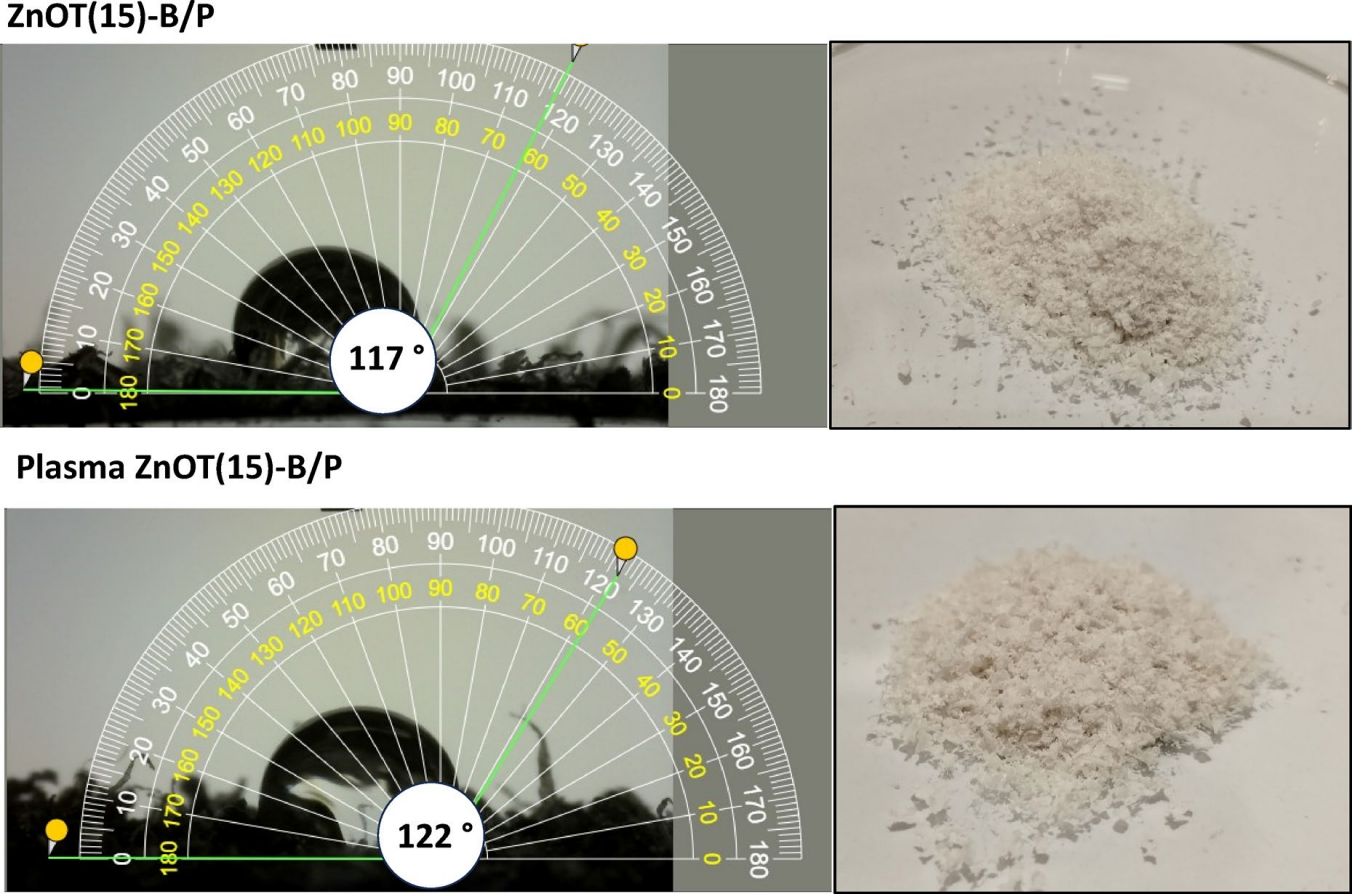
Water contact angle of ZnOT(15)-B/P before and after plasma treatment.

The surface wettability of the ZnOT(15)-B/P composite after plasma treatment was analyzed using WCA, as shown in Fig. 11. The water contact angles before and after plasma treatment were 117° and 122°, respectively. AFM revealed that the surface roughness of ZnOT(15)-B/P composite increased following plasma treatment, creating a rough texture that traps air. This enhanced roughness contributes to a greater resistance to wetting, thereby promoting super hydrophobicity. According to the Wenzel equation, surface roughness has a different effect on the contact angle based on its value; it decreases the contact angle when it is below 90° and increases it when above 90°^72^. The observed increase in water contact angle indicates enhanced water repellence and greater oil adsorption, which further improves the photocatalytic activity of the ZnOT(15)-B/P.

**Fig. 12:**
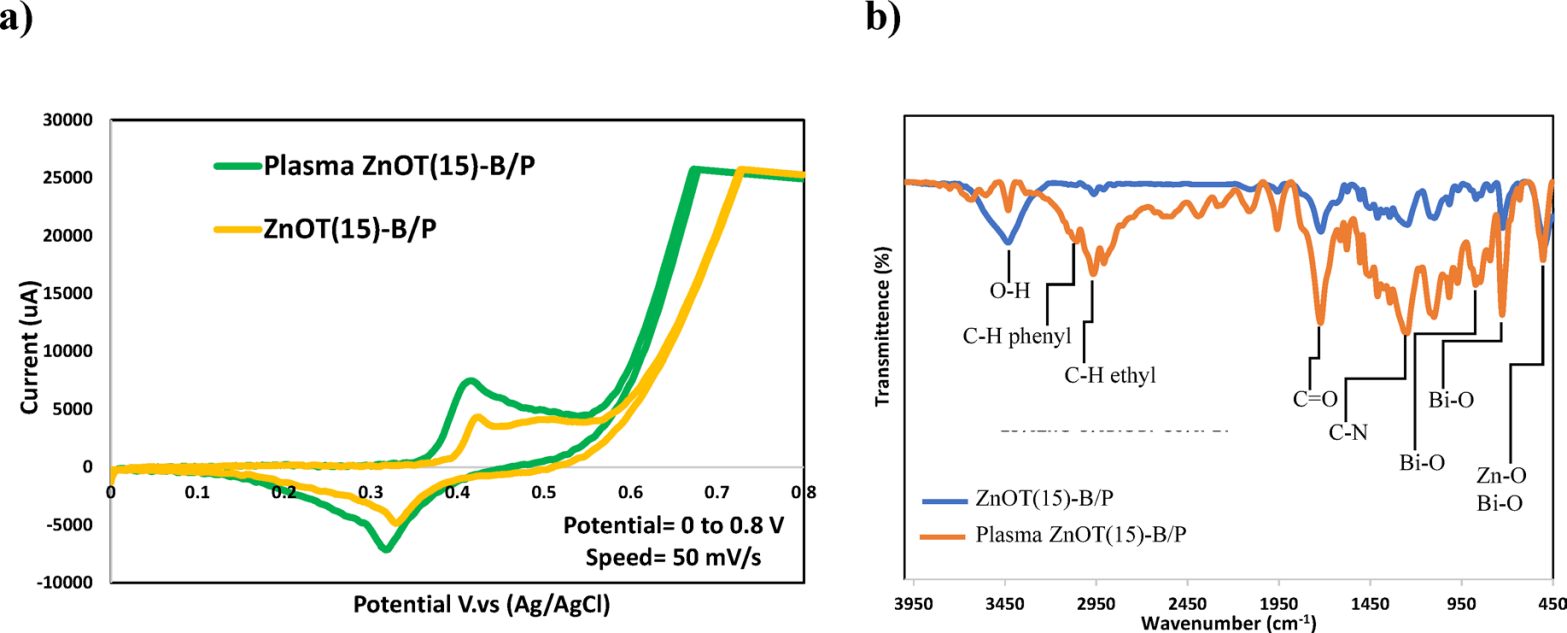
(**a**) Cyclic voltammetry and (**b**) FTIR spectra of ZnOT(15)-B/P before and after plasma treatment.

The CV analysis (Fig. 12a) reveals significant enhancements in electrochemical performance of ZnOT(15)-B/P before and after plasma treatment. The plasma-treated sample (Plasma ZnOT(15)-B/P) exhibits a higher current density and sharper redox peaks compared to the untreated material (ZnOT(15)-B/P), indicating improved charge transfer kinetics and surface activity. This enhancement can be attributed to plasma-induced modifications, such as increased oxygen vacancies, surface functionalization, and improved crystallinity, which collectively boost conductivity and active site availability. The more reversible redox behavior of the plasma-treated sample suggests superior stability and efficiency.

Figure 12b illustrates the FTIR spectra of the ZnOT(15)-B/P before and after plasma treatment. Notably, the peaks in the 3200–3950 cm^−1^ range—typically associated with the stretching vibrations of hydroxyl (O–H) groups—were reduced, aligning with the WCA analysis, which indicates that plasma treatment enhances the hydrophobicity of ZnOT(15)-B/P. Furthermore, the dark test shown in Fig. 8 suggests an increased adsorption of hexane on the plasma-treated sample, likely resulting from its enhanced hydrophobicity.

The region between 2800 and 3200 cm^−1^ corresponds to the C–H stretching vibrations. Alterations in this area may indicate structural changes in the polymer chains of PET following plasma treatment^73^. The peak at 1723 cm^−1^ typically reflects C=O stretching vibrations; variations in this peak could signal changes or the formation of carbonyl groups in PET post-treatment^74^. Additionally, the 1000–1500 cm^−1^ range encompasses bending vibrations of C–H and C–O bonds, with increased intensity of these PET-related peaks indicating that electron bombardment during plasma treatment has likely resulted in surface fragmentation, enhancing the accessibility of PET. This conclusion is further supported by AFM analysis, which confirmed increased surface roughness.

Peaks in the 450–1000 cm^−1^ range are associated with the bending and stretching vibrations of metal bonds, such as Zn–O and Bi–O. The sharpness of these peaks may suggest structural modifications of the metallic components^75–77^, arising from their increased accessibility on the surface following plasma treatment. This finding is corroborated by FESEM and AFM observations, which contribute to the improved activity of the sample in hexane degradation.

A significant transformation noted in the functional groups of the plasma-treated sample is the appearance of C–N peak^78^ on the surface, likely due to the presence of N_2_ in the air used for plasma treatment. This formation may account for the observed evacuation of hydroxyl groups from the surface.

**Fig. 13:**
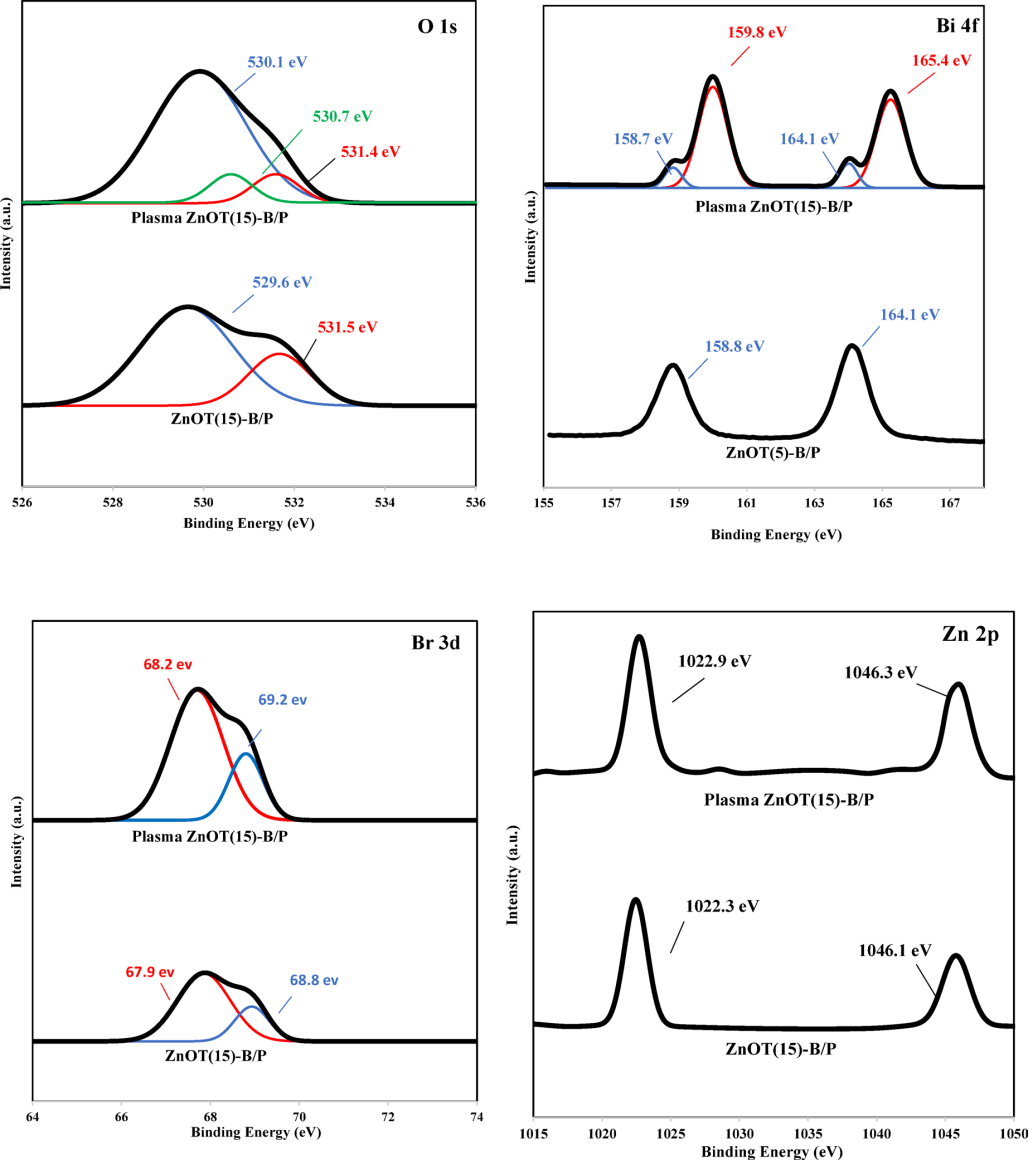
XPS spectra of O1*s*, Bi 4*f*, Br 3*d* and Zn 2*p* for ZnOT(15)-B/P before and after plasma treatment.

Figure 13 presents the O 1*s* spectra for ZnOT(15)-B/P before and after plasma treatment. Three oxygen peaks from lower binding energies to higher ones are related to lattice oxygen, oxygen vacancies, and surface-adsorbed oxygen or hydroxyl groups, respectively^79^. While the middle peak is absent in the ZnOT(15)-B/P, the plasma-treated ZnOT(15)-B/P, indicated the oxygen vacancy peak. Non-thermal plasma is a promising tool for creating oxygen vacancies on surfaces of materials^80,81^ which facilitates the electron movement from the valence band to conduction band, increasing the carrier densities (confirmed by CV analysis) and absorbing more visible light for oxidation reaction. Additionally, rich oxygen vacancies are more electropositive, preventing the recombination of electrons and holes, enhancing the life time of charge carriers^82^. The O in hydroxyl groups has lost its intensity after plasma treatment as well that is in line with FTIR and WCA, meaning evacuating OH groups from the surface of the photocatalyst during plasma treatment.

The Bi 4*f* peaks split into two, attributed to the change in the oxidation state of bismuth, resulting in the coexistence of Bi^3+^ and Bi^2+^^64,83^.

The deconvoluted Br 3d spectrum of ZnOT(15)-B/P before and after plasma treatment and surface re-oxidation shows the Br peaks shift toward higher binding energies, indicating surface modifications induced by the plasma^28^.

Zn 2*p* spectrum, shows two main peaks at 1022.28 eV and 1045.6 eV, corresponding to Zn 2*p*3/2 and Zn 2*p*1/2, respectively, indicating lattice Zn^2+^ in the ZnO wurtzite structure^84^. These peaks, with slight shifts, are also observed in plasma-treated ZnOT(15)-B/P, however, some small peaks probably due to Zn vacancy or doping of N and O has appeared in the plasma treated sample.

As we expect higher stability of inorganic materials on PET after plasma treatment, the ICP test for Bi and Zn in treated wastewater using plasma-treated 15%ZnO–5%BiOBr–80%PET exhibited 0.22 ppm and 0.1 ppm, respectively. The low amounts of metals in the treated wastewater can be attributed to the proper bonding between the components of the plasma-treated photocatalysts. This higher interaction was observed in FESEM after plasma treatment.

### Mechanism of plasma surface treatment

**Fig. 14:**
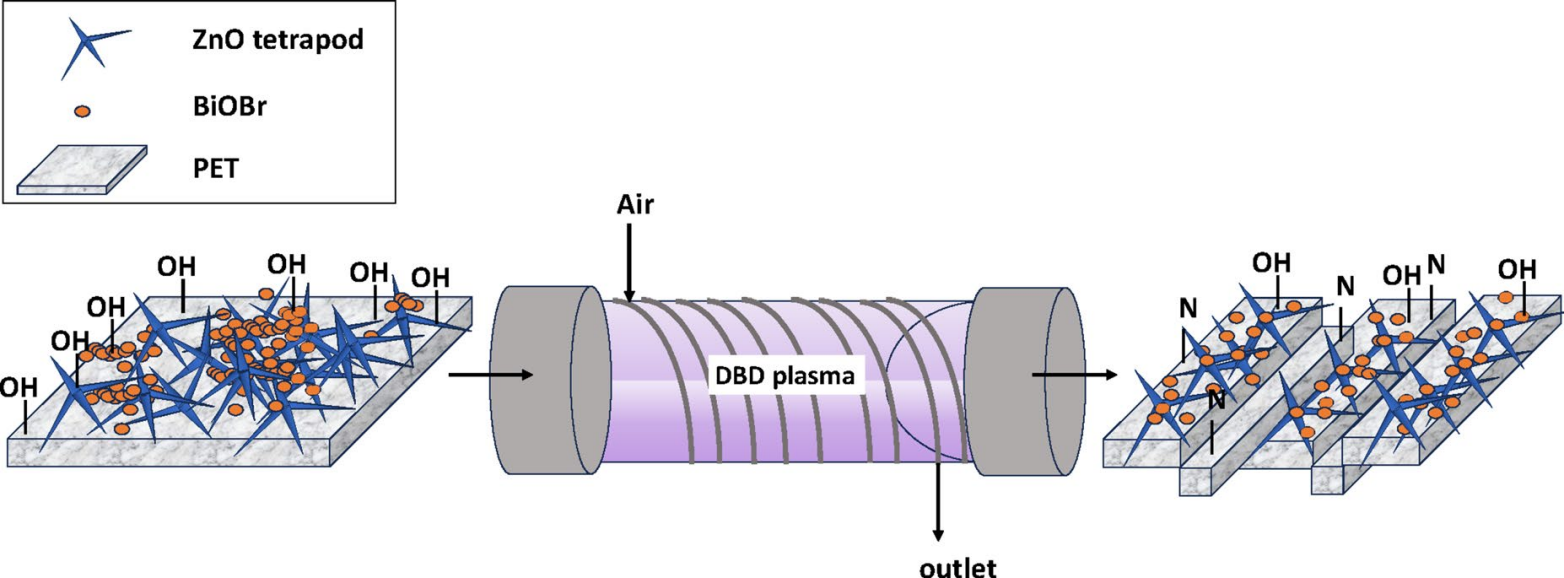
Mechanism of plasma surface treatment of ZnOT(P)-B/PET.

As indicated in Fig. 14 The mechanism of plasma surface treatment of the ZnOT(15)-B/P photocatalyst involves several interrelated processes. Initially, exposure to air plasma introduces energetic ions and electrons that interact with the surface of the photocatalyst, resulting in surface functionalization and increased roughness. This rough texture facilitates a higher surface area for hexane adsorption, promoting better contact with the photocatalyst. Concurrently, the energetic species lead to the detachment of surface particles, thus enhancing the accessibility of the active sites for photocatalytic reactions. The plasma treatment also modifies the electronic structure of the material, improving the transfer of electrons and holes, which are critical for photocatalytic efficacy. Furthermore, the treatment reduces the density of hydroxyl groups on the surface while potentially introducing nitrogen-containing functional groups, contributing to enhanced hydrophobicity and superhydrophobic characteristics of the surface. Consequently, the tailored surface morphology and altered chemical functionalities collectively favor the photocatalytic degradation of hexane by ensuring effective charge carrier separation and optimal interaction with the target molecules.

## Conclusions

In this study, we synthesized a heterostructure composed of ZnO tetrapods and BiOBr on recycled PET from drinking bottles and employed it for the photocatalytic degradation of hexane in water emulsion. Our findings indicate that the weight ratio between ZnO tetrapods and BiOBr significantly influences the photocatalyst’s performance. The optimal ratio was determined to be 5% wt. of ZnO, which resulted in strong interactions between the two components, increased surface area and porosity, enhanced adherence of the metallic compounds to PET, greater hexane adsorption, water repellence, efficient light harvesting, and a reduced recombination rate of charge carriers. This optimized photocatalyst achieved a remarkable degradation of 96.6% of hexane in water within 40 min. Further investigations demonstrated that even less active photocatalysts exhibited significantly enhanced performance in hexane degradation after surface treatment with atmospheric pressure DBD plasma. This improvement was attributed to plasma-induced surface roughening, which involves the etching away of the material and its redeposition elsewhere on the surface. This process enhances component interactions, formation of oxygen vacancies, increases hexane adsorption, enhances electrochemical properties, and ultimately leads to greater hexane degradation under visible light.

## Supplementary Information

Below is the link to the electronic supplementary material.

Supplementary Material 1

## Data Availability

The datasets generated and/or analyzed during the current study are available from the corresponding author (Somaiyeh Allahyari) on reasonable request.
