# Supplementary material for: Plasma treatment of ZnO tetrapod–BiOBr heterojunction supported on PET waste for photocatalytic degradation of oil in water
Source: Sci Rep. 2025 Dec 27;16:2903. doi: 10.1038/s41598-025-32882-2 (PMC12830707; doi:10.1038/s41598-025-32882-2)
Supplement: Supplementary file 1 — Supplementary Material 1 [file 41598_2025_32882_MOESM1_ESM.docx]

Supplementary Files

Enhanced simulated solar light-driven photocatalytic degradation of oil in water using plasma-treated ZnO tetrapod-BiOBr on recycled plastic waste

Fahimeh Nourabi ^1^, Somaiyeh Allahyari *^1^, Nader Rahemi^2^, Yogendra Kumar Mishra^3^

1. Chemical Engineering Faculty, Sahand University of Technology, P.O.Box 51335-1996, Sahand New Town, Tabriz, Iran.
2. Energy and Fuel Laboratory, Sahand University of Technology, P.O.Box 51335-1996, Sahand New Town, Tabriz, Iran.
3. Smart Materials, NanoSYD, Mads Clausen Institute, University of Southern Denmark, Alsion 2, Sønderborg, 6400, Denmark

**Table 1S. Textural properties of ZnOT(5)-B/P, ZnOT(10)-B/P, and ZnOT(15)-B/P**

| **Total pore volume (cm^3^/g)** | **Average pore diameter (nm)** | **BET surface area (m^2^/g)** | **Catalyst** |
| --- | --- | --- | --- |
| 0.095 | 13.3 | 28.5 | **ZnOT(5)-B/P** |
| 0.08 | 11.5 | 28.4 | **ZnOT(10)-B/P** |
| 0.065 | 13.2 | 19.9 | **ZnOT(15)-B/P** |


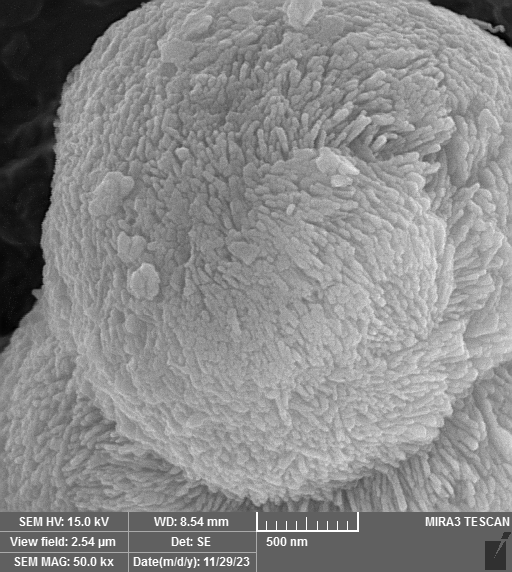


**Figure 1S. FESEM micrographs of BiOBr.**

**Figure 2S. XRD patterns of ZnOT(5)-B/P, ZnOT(10)-B/P, and ZnOT(15)-B/P**

**Figure 3S. Adsorption-desorption isotherms and pore size distribution (inset) of ZnOT(5)-B/P, ZnOT(10)-B/P, and ZnOT(15)-B/P**

| **a)** | **b)** |
| --- | --- |

**Figure 4S. a) TGA and b) DTG analysis of PET and ZnOT(5)-B/P**

**Figure 5S. a) UV spectrum of different hexabne in water mixtures and b) its calibration curve.**

**Figure 6S. Effect of wastewater pH on photocatalytic degradation of hexane using ZnOT(5)-B/P**

**Figure 7S. Effect of hexane concentration on photocatalytic degradation of hexane using ZnOT(5)-B/P**

**Figure 8S. Effect of photocatalyst loading on photocatalytic degradation of hexane using ZnOT(5)-B/P**

**Figure 9S. Reusability of ZnOT(5)-B/P in photocatalytic degradation of hexane**
